# The influence of marital status on the survival of patients with Hodgkin lymphoma

**DOI:** 10.18632/oncotarget.16879

**Published:** 2017-04-06

**Authors:** Fangfang Wang, Xiaoyan Xie, Xiaoming Yang, Guoqing Jiang, Jian Gu

**Affiliations:** ^1^ Department of Hematology, Clinical Medical College of Yangzhou University, Subei People's Hospital of Jiangsu Province, Yangzhou, China; ^2^ Medical Department, Clinical Medical College of Yangzhou University, Subei People's Hospital of Jiangsu Province, Yangzhou, China; ^3^ Department of Hepatobiliary and Pancreatic Surgery, Clinical Medical College of Yangzhou University, Subei People's Hospital of Jiangsu Province, Yangzhou, China

**Keywords:** Hodgkin lymphoma, marital status, survival analysis, surveillance, epidemiology and end results

## Abstract

Marital status is viewed as an independent prognostic factor for survival in various cancers. But, little is known about the relationship between marital status and Hodgkin lymphoma (HL) outcomes. To investigate the impact of marital status on the survival of patients with HL, we identified 37884 cases from 1988 to 2013 in the Surveillance, Epidemiology and End Results (SEER) database. The Kaplan–Meier method and multivariate Cox regression model were used for analyzing the influence of marital status on cause-specific survival (CSS). We found patients in widowed group had a higher proportion of women and a higher incidence of older (>60 years) patients; all of these parameters were found to be statistically significant in within-group comparisons. Marital status was demonstrated to be an independent prognostic factor. Widowed individuals were at greater risk of cancer specific mortality relative to other groups. Similar associations in subgroup analyses were observed according to SEER stage. In conclusion, widowed patients suffered survival disadvantages relative to other groups, and marital status had significant prognostic value in HL.

## INTRODUCTION

Hodgkin lymphoma (HL) is a rare cancer of the lymphatic system [1]. It accounts for 10% of all lymphomas and less than 1% of all cancers diagnosed in United States (US) annually [2]. Approximately 8500 new patients (3,710 females and 4,790 males) will be diagnosed with HL and 1120 (480 females and 640 males) will die of the disease in the US in 2016 according to projections [2, 3]. In the 1960s, the 5-year survival rate for HL was less than 10% [4]. Because of advances in treatment, survival has improved; the reported 5-year survival rate for patients with HL during the years 2000-2004 was 85.2% [5]. However, there are differences in patient survival related to the tumor's histology and its stage at diagnosis.

Marital status has been investigated a number of cancers with results often showing significant differences in incidence, disease characteristics and survival as a function of marital status [6]. A larger population-based study of data indicated that unmarried patients, including those who are widowed, are at significantly greater risk of presentation with metastatic cancer, undertreatment and cancer-related death than patients who are married [7]. Although numerous studies have measured the relationship between marital status and cancer incidence and survival, little is known about the relationship between marital status and HL outcomes. We used the Surveillance, Epidemiology and End Results (SEER) database to study the impact of marital status on HL cause-specific survival (CSS) and the survival disparities between married and unmarried individuals.

## RESULTS

### Patient baseline characteristics

A total of 37884 eligible patients were identified from 1988 to 2013, including 20633 male and 17251 female patients. Of these, 1610 were widowed, 16728 were married, 17034 had never married and 2512 were divorced /separated in our study. Patients in the widowed group had the following characteristics: the highest proportion of women; a greater prevalence of elderly patients (median 76 years). Both of these parameters differed significantly in within-group comparisons (P<0.001). The incidence rate of elderly patients (>60 years) in widowed group was significantly higher than that of the younger widowed group (87.6% vs. 12.4%), while the incidence rate among patients aged >60 years in married group, never married group and divorced/separated group was considerably lower than among younger patients. Patient demographics and pathological features are summarized in Table 1.

**Table 1:** Baseline demographic and tumor characteristics of patients in the SEER database

Characteristic	Total	Widowed	Married	Never married	Divorced/separated	P
	(n=37884)	(n=1610) N (%)	(n=16728) N (%)	(n=17034) N (%)	(n=2512) N (%)	
**Sex**						<0.001
Male	20633	381(23.7)	9420(56.3)	9588(56.3)	1224(49.5)	
Female	17251	1229(76.3)	7308(43.7)	7446(43.7)	1268(50.5)	
**Race**						<0.001
White	31554	1401(87.0)	14633(87.5)	13442(78.9)	2078(82.7)	
Black	4296	138(8.6)	1199(7.2)	2610(15.3)	349(13.9)	
Other*	2034	71(4.4)	896(5.4)	982(5.8)	85(3.4)	
**Age^#^**		76	43	24	48	<0.001
≤60	31554	200(12.4)	12945(77.4)	16465(96.7)	1944(77.4)	
>60	6330	1410(87.6)	3783(22.6)	569(3.3)	568(22.6)	
**Histotype**						<0.001
LR	1218	64(4.0)	648(3.9)	403(2.4)	103(4.1)	
MC	5189	371(23.0)	2496(14.9)	1891(11.1)	431(17.2)	
LD	501	55(3.4)	239(1.4)	165(1.0)	42(1.7)	
NS	22807	604(37.5)	9600(57.4)	11279(66.2)	1324(52.7)	
LP	1590	52(3.2)	774(4.6)	652(3.8)	112(4.5)	
Unknown	6579	464(28.8)	2971(17.8)	2644(15.5)	500(19.9)	
**Ann Arbor stage**						<0.001
I/II	22179	763(47.4)	10032(60.0)	10016(58.8)	1368(54.5)	
III/IV	14082	736(45.7)	5948(35.6)	6360(37.3)	1038(41.3)	
Unknown	1622	111(6.9)	748(4.5)	657(3.9)	106(4.2)	
**Year of diagnosis**						<0.001
1988-1996	7234	327(20.3)	3333(19.9)	3112(18.3)	462(18.4)	
1997-2005	14575	660(41.0)	6608(39.5)	6265(36.8)	1042(41.5)	
2006-2013	16075	623(38.7)	6787(40.6)	7657(45)	1008(40.1)	

*Other includes American Indian/Alaska native, Asian/Pacific Islander, and unknown.

^#^-Median age.

### Effect of marital status on CSS in the SEER database

**Figure 1:**
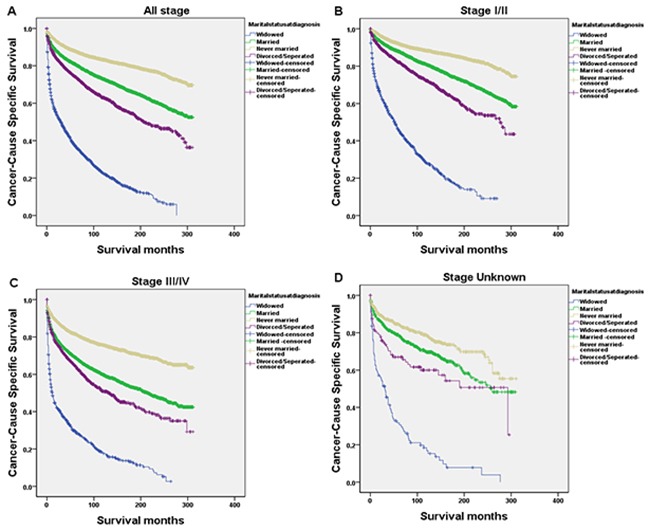
Survival curves in HL patients according to marital status **(A)** Overall (Stage I-IV and Unknown); χ2=4553.501, P<0.0001; **(B)** Stage I/II; χ2=2630.949, P<0.0001; **(C)** Stage III/IV; χ2=1632.126, P<0.0001; **(D)** Stage Unknown; χ2=251.090; P<0.0001.

We performed Kaplan-Meier analysis to calculate CSS. The survival difference among the different marital status was significant according to the univariate log-rank test (P<0.001). The overall 5-year CSS was 37.4% in the widowed group, 80.9% in the married group, 87.9% in the never married group and 74.2% in the divorced/separated group; thus, widowed patients had a significantly inferior CSS as compared with the other groups (Figure 1A). In addition to marital status, male sex (P<0.001), Black ethnicity (P<0.001), old age (P<0.001), lymphocyte-depleted (LD) histotype (P<0.001), Ann Arbor stage III/IV (P<0.001) and early diagnosis (P<0.001) were found to be significant risk factors for poor survival in univariate analysis (Table 2). These variables were validated as independent prognostic factors when multivariate analysis with Cox regression was performed, as follows: sex; age; histotype; Ann Arbor stage; year of diagnosis; and marital status (widowed, 1.317, 95% confidence interval [CI] 1.202-1.444; married, HR 0.711, 95%CI 0.661–0.765; never married, HR 0.636, 95%CI 0.587–0.688).

**Table 2:** Univariate and multivariate survival analysis regarding evaluation of the influence of marital status on HL cause-specific survival in the SEER database

Variable	5-year CCS	Univariate analysis		Multivariate analysis	
		Log rank χ^2^ test	P	HR(95%CI)	P
**Sex**		168.758	<0.001		
Male	79.9%			1.430(1.369-1.495)	<0.001
Female	83.8%			Reference	
**Race**		36.623	<0.001		
White	81.9%			1.004(0.907-1.111)	0.942
Black	79.1%			1.440(1.282-1.616)	<0.001
Other*	83.9%			Reference	
**Age**		11542.410	<0.001		
≤60	88.9%			Reference	
>60	45.4%			5.479(5.202-5.770)	<0.001
**Histotype**		2218.610	<0.001		
LR	84.8%			Reference	
MC	73.3%			1.446(1.280-1.633)	<0.001
LD	48.7%			2.836(2.415-3.331)	<0.001
NS	86.7%			0.686(0.610-0.771)	<0.001
LP	91.4%			0.616(0.516-0.734)	<0.001
Unknown	70.3%			1.549(1.372-1.749)	<0.001
**Ann Arbor stage**		1445.803	<0.001		
I/II	87.9%			Reference	
III/IV	72.3%			2.003(1.919-2.092)	<0.001
Unknown	76.6%			1.579(1.437-1.735)	<0.001
**Year of diagnosis**		75.552	<0.001		
1988-1996	78.8%			1.445(1.359-1.536)	<0.001
1997-2005	81.6%			1.138(1.120-1.249)	<0.001
2006-2013	83.7%			Reference	
**Marital status**		4553.501	<0.001		
Widowed	37.4%			1.317(1.202-1.444)	<0.001
Married	80.9%			0.711(0.661-0.765)	<0.001
Never married	87.9%			0.635(0.587-0.688)	<0.001
Divorced/separated	74.2%			Reference	

*Other includes American Indian/Alaska native, Asian/Pacific Islander, and unknown.

### Subgroup analysis of the effects of marital status according to Ann Arbor stage

We further explored the effects of marital status on survival regarding tumor stage. We observed that marital status was still an independent prognostic factor concerning tumor stage, both in univariate and multivariate analysis (P<0.001). In addition, patients in the widowed group always had the lowest survival rate. Widowed patients had a clear reduction in 5-year CSS as compared with the other groups with stage I/II (45.8% vs. 87.9%, 92.1%, 82.1%; HR 3.834, 95% CI 3.353-4.385, P<0.001), stage III/IV (29.6% vs. 69.1%, 81.6%, 64.5%; HR 2.728, 95%CI 2.411-3.086, P<0.001) and stage Unknown (30.6% vs. 78.2%, 84.4%, 65.6%; HR 3.160, 95%CI 2.188-4.564, P<0.001) cancer (Table 3, Figure 1B-1D). Moreover, the 5-year CSS in the never married group was the highest, with an increase of 2-3 times relative to the widowed groups (Table 3).

**Table 3:** Univariate and multivariate analysis of the effects of marital status on HL cause-specific survival based on different cancer stages

Variable	5-year CCS	Univariate analysis		Multivariate analysis	
		Log rank χ^2^ test	P	HR(95%CI)	P
**Ann Arbor stage**					
**Stage I/II**					
**Marital status**		2630.949	<0.001		
Widowed	45.8%			3.834(3.353-4.385)	<0.001
Married	87.9%			0.654(0.585-0.730)	<0.001
Never married	92.1%			0.365(0.324-0.410)	<0.001
Divorced/separated	82.1%			Reference	
**Stage III/IV**					
**Marital status**		1632.126	<0.001		
Widowed	29.6%			2.728(2.411-3.086)	<0.001
Married	69.1%			0.804(0.727-0.889)	<0.001
Never married	81.6%			0.434(0.391-0.483)	<0.001
Divorced/separated	64.5%			Reference	
**Unknown**					
**Marital status**		251.090	<0.001		
Widowed	30.6%			3.160(2.188-4.564)	<0.001
Married	78.2%			0.701(0.504-0.976)	0.035
Never married	84.4%			0.495(0.351-0.698)	<0.001
Divorced/separated	65.6%			Reference	

## DISCUSSION

Our study is the largest to examine survival disparities as a function of marital status in HL population. Using the SEER database to investigate the relationship between marital status and survival, we found that marital status was an independent prognostic factor for patients with various Ann Arbor disease stages. Widowed patients had a significantly poorer CSS than married counterparts. In multivariate analysis, the risk for widowed patients persisted even after adjusting for sex, age, race, histotype, year of diagnosis and SEER stage. In addition, interaction was found between sex, age, year of diagnosis and marital status for these prognostic factors. The patients who were older (>60 years), more tumors at stage III/IV, early year of diagnosis or the inferior LD histotype, had the worst 5-year CSS.

It has been reported that unmarried individuals, especially widowed patients, have a lower CSS than married ones [8, 9]. This also appears to be the case in our study, because marital status emerged as a statistically significant factor in both univariate analyses and in multivariate models. Regarding the widowed populations, a trend regarding increased mortality was less clear than in married populations. One of the potential reasons for the lower CSS in widowed patients is delayed diagnosis of patients with advanced tumor stages. In our study group, survival of patients with stage III/IV in widowed group decreased sharply in the first 2 years (Figure 1C); they had a worse 5-year CSS as compared with all the other groups (P<0.001).

The relationship between marital status and survival can be explained hypothetically by psychosocial factors. A cancer diagnosis can be more psychologically distressing than other diagnoses [10]. Stress has been shown to have a more direct effect on physical health [11, 12]. Psychological stress could in turn lead to more risky health-related behavior and poor sleep, thus adversely affecting general physical health status [13]. And some studies even suggest adverse effects regarding tumor growth [14]. It has been proposed that decreased psychosocial support and psychological stress alter immune function and contribute to tumor progression and mortality [15, 16]. Patients who are married display less distress and anxiety than their unmarried counterparts after a diagnosis of cancer; this is because a partner can share the emotional burden and provide appropriate social support [17]. A study of the association between partner support and psychological distress among prostate cancer survivors showed that married prostate cancer survivors with high partner support reported significantly lower levels of psychological distress than unmarried survivors and married survivors with low partner support [18]. Additionally, psychological stress may cause poorer adherence to treatment regimens [13]. It is possible that married individuals receive better treatment from hospitals than unmarried individuals. A meta-analysis suggests that marriage positively influences adherence to treatment, partly through the partner's support [19]. Otherwise, unmarried patients may have more emotional burden and experience a lack of support from society and the spouse. Increased psychological stress may worsen cancer outcome [18, 20].

Interestingly, our study revealed that the 5-year CSS in the never-married group was better than in the married group, although survival benefits associated with married patients are supported by many studies. This may be the result of the following factors: good physical health because these patients are usually young at the time of diagnosis; the advantage of early disease stage; the protective effect of parental care; or extensive social support.

In the present study, the proportion of widowed elderly patients (>60 years) was extremely high (87.6%); this suggested that the inferior CSS of widowed patients may be correlated with age. The poor survival of elderly widowed women who might have poorer overall physical health at time of diagnosis may be driven by poorer socioeconomic status, decreased access to healthcare and loss of social support. The elderly widowed female patient's loss of social support or their inability to cope with stress may lead to increased mortality [21].

This study has several obvious strengths. The database is an authoritative source of information on cancer incidence and survival in the US, the time-span covered was rather large, and the patient and tumor information collected was very comprehensive. However, the present study had several limitations. We hypothesized that psychosocial factors may be the main reasons for poor survival of widowed patients; however, the patient's psychological condition at time of diagnosis was not known. Perhaps there were undiagnosed cases of mental disease in our sample. We were unable to adjust for pre-widowhood disease status, which had some impact on the CSS. Moreover, it has not been possible to distinguish between never-married and individuals who cohabit. Some patients who were classified as never married may have been cohabitating. In addition, some patient's marital status may have changed during the therapeutic process, which would have interfered with analytical results.

In conclusion, to the best of our knowledge this is the first study that has reported on the association between martial status and survival in patients with HL. Our study suggests that there was a strong positive relationship between these two factors; widowed patients had a significantly higher risk of mortality. Psychosocial factors may be one of the primary reasons for poor survival in widowed patients. More social support and care should be provided for these patients. Incorporating information about marital status into the design of intervention programs may help better target potential beneficiaries among widowed patients with HL.

## MATERIALS AND METHODS

### Data source

Patient demographics, disease characteristics, survival data and marital status were obtained using the SEER program, which is sponsored by the National Cancer Institute [7]. The current SEER database consists of 18 population-based cancer registries acquired from 1988 to 2013, which represent approximately 28% of the US population [22]. It is made available for researchers to study the relationship between marital status and the survival outcomes of patients with cancer [6–8, 23–26].

We limited the histological type of HL using the International Classification of Diseases for Oncology third edition (ICD-O-3) morphology codes (LR: 9651; MC: 9652; LD: 9653, 9654, 9655; LP: 9659; NS: 9663, 9664, 9665, 9667; and Unknown: 9650).

### Patient selection and data extracted

Using the SEER-stat software (SEER*Stat 8.1.5), we searched for patients diagnosed between 1988 and 2013 with HL and a known marital status. Patient exclusion criteria were as follows: (1) they had more than one primary cancer but HL was not the first one; (2) they had unknown marital status; and (3) the cause of death was unknown or their survival time was unknown. We obtained data on patient gender, age, race, histotype, Ann Arbor stage, year of diagnosis and marital status from the SEER database.

### Statistical analysis

Within the SEER database, the stage was established according to the 1983^+^ Ann Arbor classification criteria. Marital status was coded as married, never married, widowed, divorced and separated. We assigned the separated and divorced patients into the divorced/separated group in the present study [27].

Chi-squared tests were conducted to examine differences in the frequency of patient baseline characteristics among marital status. Differences in survival were estimated using the Kaplan-Meier method. We assessed differences in CSS by gender, age, race, histotype, Ann Arbor stage, year of diagnosis and marital status using the log-rank test or the multivariate Cox regression model. All of the statistical analyses were performed using the statistical software package SPSS for Windows, version 22 (IBM Corp, Armonk, NJ, USA). Statistical significance was set at a two-sided P value < 0.05.

## Abbreviations

HL: Hodgkin lymphoma
SEER: Surveillance, Epidemiology and End Results
CSS: cause-specific survival
US: United States
HR: hazard ratio
CI: confidence interval
NS: nodular sclerosis
LD: lymphocyte-depleted
LR: lymphocyte-rich
MC: mixed cellularity
LP: lymphocyte-predominant
ICD-O-3: International Classification of Diseases for Oncology third edition.
